# Study on Thermal Stability and Fatigue Properties of SBS/CNT-Modified Asphalt Sealant

**DOI:** 10.3390/polym15193968

**Published:** 2023-10-01

**Authors:** Yafeng Gong, Shuzheng Wu, Haipeng Bi, Lihua Tian

**Affiliations:** College of Transportation, Jilin University, Changchun 130025, China; gongyf@jlu.edu.cn (Y.G.); wusz20@mails.jlu.edu.cn (S.W.); tianlh@jlu.edu.cn (L.T.)

**Keywords:** sealant, SBS, CNTs, high-temperature performance, anti-aging properties

## Abstract

Carbon nanotubes (CNTs) and styrene–butadiene–styrene (SBS) are used as reinforcing modifiers in asphalt sealants due to their excellent properties, which can effectively improve the internal structure of the sealant and enhance its mechanical properties. Based on this background, two SBS/CNT-modified asphalt sealants were identified and selected by the orthogonal experimental method and compared with two commercially available sealants. The softening point, flow value, multi-temperature frequency scan test, and multiple stress creep recovery test were used to study the high-temperature rheological properties and aging resistance of four types of sealants. The overall evaluation shows that the proportion of the sealant compound’s preparation material is 1% by weight of CNT doping, 5% by weight of SBS doping, and 5% by weight of furfural-extracted-oil doping. The results show that the addition of SBS and CNTs more significantly improves the fatigue resistance of the sealants. With the CAM model, C1.0S5F5 reflects a better relaxation property, which better avoids secondary cracking after the construction of the sealant. With the Burgers model, C1.0S5F5 shows excellent deformation resistance under heavy traffic conditions. In summary, conventional performance indicators, such as the softening point and flow value of SBS/CNT-modified asphalt sealants, can meet the specification requirements and show good high-temperature stability and anti-aging properties compared to commercially available sealants.

## 1. Introduction

Cracks are a common disease of asphalt concrete pavements. During road use, cracks are affected by factors such as temperature, precipitation, and loading. Cracks can develop into frost swelling and mineral spalling during stretching, destroying the structural integrity and service life of roads [1,2,3,4]. Therefore, the timely repair of cracks is very significant. Asphalt pavement crack repair materials can be divided into hot irrigation sealants, cold irrigation sealants, and special sealant materials. The cold-irrigation sealant-type material can be applied at room temperature; however, its failure rate is high, so it is often used for emergency repairs in harsh environments [5]. Specialized sealant materials offer superior performance but are more costly and complex to work with. Hot irrigation sealants are less expensive, perform much better than cold irrigated materials, and are commonly used crack treatment materials [6]. Therefore, the use of hot irrigation sealants to seal cracks can effectively prevent surface water and debris from entering the pavement structure and restore the load-bearing capacity of the pavement to a certain extent.

Hot asphalt is a traditional hot irrigation material with some bonding and sealing properties, but its performance is poor, with the elasticity and durability able to be improved [7]. A survey found that the temperature difference for an area with a sealant life of only about 1 year means the sealant needs to be re-patched, as the performance of the sealant is closely related to the construction of the road and the maintenance funds invested into the situation; this phenomenon has caused a huge waste of manpower and funds [7,8,9]. Modified asphalt sealant materials solve this problem. Many researchers worked on modified asphalt sealants [10]. With asphalt as the matrix, by adding modifiers to improve the high- and low-temperature stability, anti-aging, elasticity, and other properties of sealant materials. At present, hot irrigation sealants mostly use rubber, polymer, and some inorganic materials to improve the strength of sealants. For example, styrene–butadiene–styrene (SBS) is currently the most widely used asphalt modifier. But rubber and polymer still have shortcomings in performance and engineering applications. Cao et al. [11] exposed two types of sealants directly to the natural environment for 8 months to study the aging characteristics and the aging mechanism of the sealants. The surface of the sealants hardened, became brittle, and cracked after aging; the polymer particles degraded; and the light component of the asphalt matrix gradually transformed into a granular asphaltene. Kim et al. [12] modified solvent deasphalting (SDA) residual oil with oil, SBS, and rubber powder, and the results showed that the addition of rubber powder reduced the adhesion of the sealants. The problems of low bonding, a short fatigue life, and a lack of aging resistance in the research of modified asphalt sealants have limited the promotion and engineering application of sealants. To address these problems, it is very essential to find a new modifier to be applied to a crack sealant to optimize its performance and increase its durability.

Carbon nanotubes (CNTs), long hollow cylinders of graphene, have an enormous specific surface area and are stronger, stiffer, and tougher than even most metallic materials [13]. The fine scale of this fibrous structure also allows it to form a complex mesh structure with only small additions to the asphalt, which improves the strength and toughness of the asphalt [14]. In addition, CNTs help to improve the fatigue and aging resistance of modified asphalt [15]. Ibrahim Amin et al. [16] studied the effect of multi-walled carbon nanotubes (MWCNTs) on the rheology of asphalt by controlling the additive content using MWCNTs as a modifier and found that the temperature sensitivity of asphalt increased when the content of MWCNTs was increased from 1% to 3% against the empirical and rheological properties of asphalt. Wang et al. [17] used CNTs as modifiers to evaluate the decay of the physical properties of modified asphalt after aging using participation in the needle penetration ratio, the softening point increment, the residual ductility ratio, and the viscosity aging index. The results showed that CNTs reduced the degree of attenuation of the physical properties of the modified asphalt. In addition, the rheological parameters obtained from the rheological tests showed that the addition of CNTs reduced the increase in the complex modulus and the decay in the phase angle of modified asphalt after aging. These results showed that CNTs have some anti-aging effects and can prevent the hardening of asphalt to a certain extent. CNTs act as a reinforcement at the interface between the polymer phase and the asphalt phase, resulting in a dense network structure and influencing the molecular ground motion in the asphalt, thus giving the modified asphalt better mechanical properties [18].

As an important means of road maintenance, sealants are subject to complex vehicle loads and direct exposure to the natural environment during road use, which requires them to be able to adapt to actual road conditions [6]. Therefore, it is of great practical engineering importance to explore the research methods used for the performance of sealants. Despite the existence of current specification standards for the study of the performance of sealants, some researchers believe that the specification standards do not adequately reflect the field performance of sealants and can only be used as an empirical standard for conventional performance indicators [19]. In recent years, the characterization of asphalt materials has gradually shifted from traditional empirical indicators to rheological indicators, and the study of rheological properties based on classical viscoelasticity theory has provided an important basis for the evaluation of the properties of asphalt materials [20]. Yang et al. [21] and Ozer et al. [22] carried out field validation studies on sealants’ performance and found a strong correlation between the rheological index and pavement sealants’ performance. Therefore, the study of rheological properties based on the classical viscoelastic mechanics theory provides an important basis for the performance evaluation of asphalt materials.

In summary, to address the current situation that the aging performance and fatigue performance of asphalt pavement sealants still need to be improved, this paper uses CNTs as the main additive to prepare modified asphalt sealants and compare the performance with two commercially available sealants. The high-temperature stability and aging resistance of SBS/CNT-modified asphalt sealants were investigated by a softening point test, flow value test, frequency scan test, and multiple stress creep recovery test. The main curve of the complex shear modulus was fitted by the CAM model. The creep process of the sealants was fitted by the Burgers model to better understand the viscoelastic intrinsic property of the modified asphalt sealants.

## 2. Materials and Methods

### 2.1. Raw Materials and Preparation of Modified Asphalt Sealants

In this study, 90# road petroleum asphalt was used, and its performance index is shown in Table 1. CNTs have a positive effect on the heat resistance, cold resistance, shear resistance, and strength of asphalt materials. In this paper, carbon nanotubes (CNTs) with 99% purity were provided from Shenzhen Suiheng Technology Co., Ltd. (Shenzhen, China), and their performance index is shown in Table 2.

**Figure 1:**
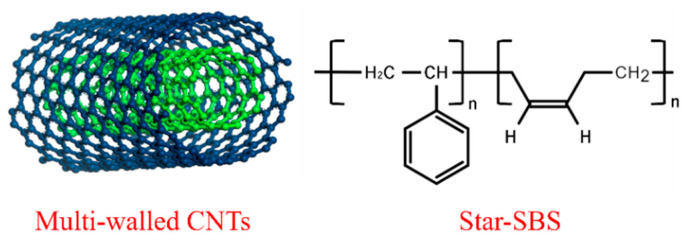
Schematic representation of CNTs and SBS molecules.

SBS interacts well with asphalt and is able to form a cross-linked network within it, thereby improving the rheological and mechanical properties of the asphalt for higher or lower temperatures. The molecular structure is shown in Figure 1. The selected SBS modifier was Star SBS, manufactured by Yanshan Petrochemical Co., Ltd. (Beijing, China). The performance indicators are shown in Table 3. The addition of saturation-rich substances as compatibilizers in the production of the sealant improved the solubility of the SBS modifier in the asphalt and enhanced the modification effect. In this study, furfural-extracted oil (FEO) was used as a compatibilizer, which, on the one hand, introduced small molecules into the asphalt system to optimize the low-temperature elasticity of the sealants, and on the other hand, improved the hardness of the sealants.

**Figure 2:**
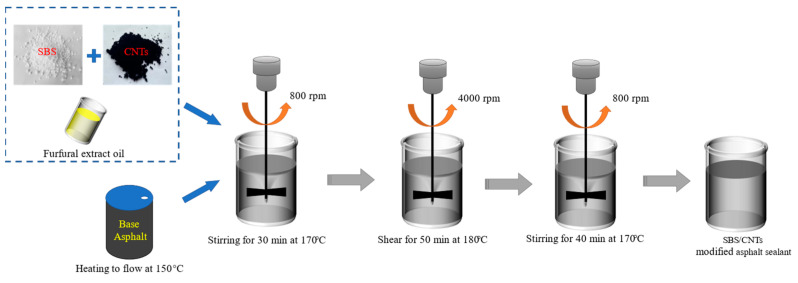
Diagram of the preparation process.

Based on preliminary experimental research, the optimum preparation process for the sealant was determined as shown in Figure 2. The substrate asphalt was heated to a fluid state and the modifier and compatibilizer were added. First, the mixed asphalt was placed in an electric mixer and stirred at a low speed (800 rpm) for 30 min at 170 °C to fully disperse it. Subsequently, the modified asphalt was subjected to high-speed mixing (5000 rpm) for 60 min at 180 °C. Finally, it was cooled down to a temperature of 170 °C and developed with stirring at 800 rpm for 40 min. The SBS/CNT-modified asphalt sealant was finally obtained.

As shown in Figure 2, the CNTs and SBS were doped under selected optimal preparation process conditions to prepare the sealant. According to the principle of orthogonal experiment design, a three-factor, three-level orthogonal experiment was designed using the CNT dosage, SBS dosage, and furfural-extracted oil dosage as factors. The factor levels were set based on pre-experiments and previous studies [1]. The designs of the factors and protocols for the orthogonal experiment are shown in Table 4 and Table 5.

In addition, two commercially available sealants were purchased for performance comparison with the prepared sealants, and the two commercially available sealants were named MS1 and MS2, the main components of which were asphalt and rubber powder. The difference was that the polymer modifier of MS1 was SBS only and the polymer modifier of MS2 was ethylene vinyl acetate copolymer (EVA).

### 2.2. Experimental Methods

#### 2.2.1. Conventional Tests

**Figure 3:**
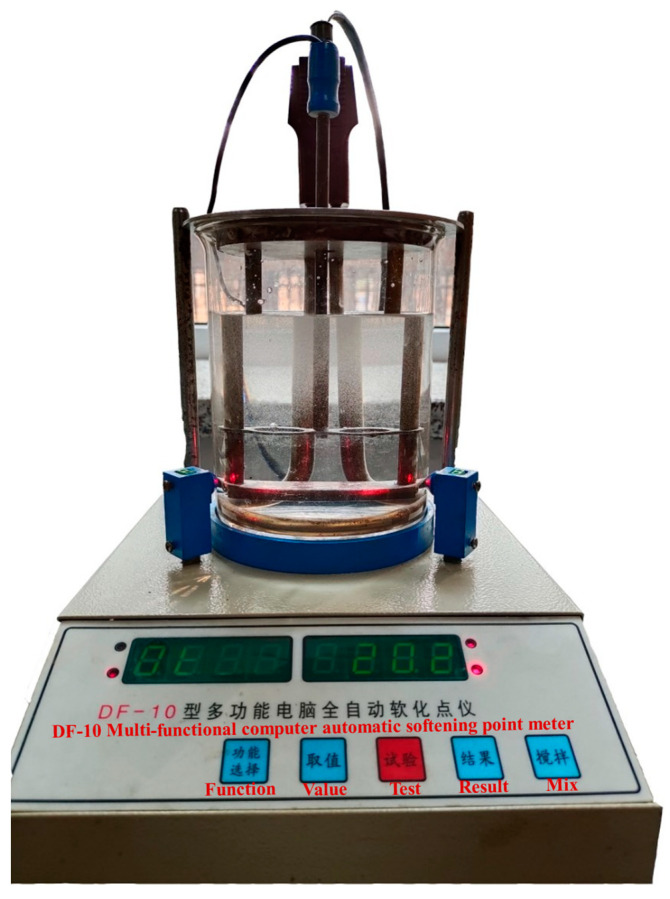
Softening point test.

**Figure 4:**
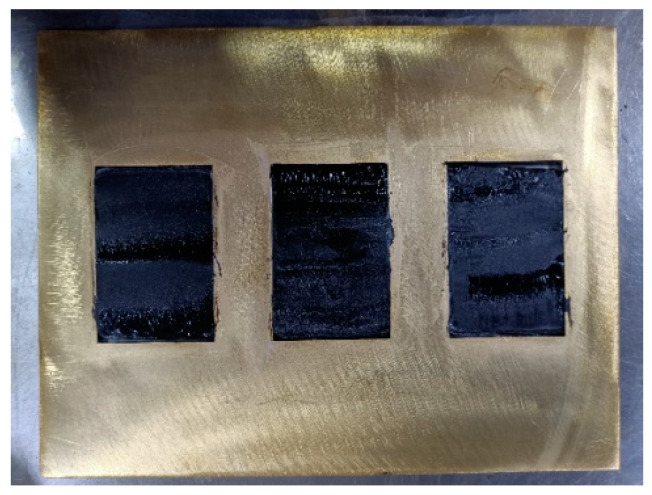
Flow value test.

A low heating temperature makes it difficult to fully fill cracks due to the lack of liquidity of the sealants, while a high heating temperature leads to the accelerated aging of the sealant. During the construction process, the pouring of the sealants often lags behind their slotting and clearing, which prolongs their heating time and makes them age. It is essential to study the viscosity of sealants at different temperatures and to simulate aging at construction temperatures. The construction temperature of sealants is between 180 °C and 200 °C. Therefore, the recommended construction temperature was used as the aging temperature. The aging of asphalt was simulated using the rotating thin film oven (RTFO) test. The sealants were stirred and heated at this temperature for 4 h. The softening point and flow value of the sealants were also tested before and after aging, as shown in Figure 3 and Figure 4.

#### 2.2.2. MSCR Tests

**Figure 5:**
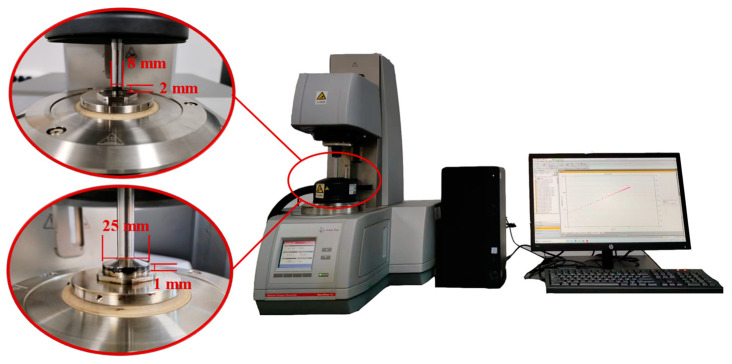
Anton Paar MCR 102 dynamic shear rheometer.

The multiple stress creep recovery (MSCR) test is a method proposed by the US Federal Highway Administration to evaluate the high-temperature rutting resistance of asphalt [23]. This test was conducted using a dynamic shear rheometer (DSR, shown in Figure 5) to apply constant stress to the specimen for 1 s per loading cycle, with the specimen recovering part of the creep deformation when the loading was stopped and the non-recoverable part being added to the next cycle of loading. This loading method simulates the vehicle action of actual pavement loading and unloading, and can well reflect the force–deformation characteristics of sealants under coupled dynamic load–temperature action [24].

The multiple stress creep recovery (MSCR) test was performed on the sealants before and after aging according to ASTM D7175 [25] to obtain the creep recovery rate *R* and the unrecoverable creep compliance $J_{nr}$. The test temperature was 60 °C, the diameter parallel plate was 25 mm with a 1 mm gap, and test cycles were repeated 10 times at 0.1 kPa and 3.2 kPa stress levels.

#### 2.2.3. Frequency Sweep Test

The frequency sweep test is one of the main test methods for testing the viscoelastic parameters of asphalt and reflects the viscoelastic response of the sealant under stress through the results of data, such as the complex shear modulus *G* and the phase angle *δ*. The testing equipment used was the Anton Paar MCR 102 dynamic shear rheometer. The frequency sweep test was carried out according to ASTM D7175 to obtain the complex shear modulus and the phase angle of the sealant. The test samples were not considered for aging. The test temperatures were 20 °C, 30 °C, 40 °C, 50 °C, and 60 °C. The loading frequency at each temperature was 0.1 to 100 Hz, and the strain was controlled to be within the linear viscoelastic strain range.

## 3. Results and Discussion

### 3.1. High-Temperature Performance Analysis Based on the Softening Point and the Flow Value

**Figure 6:**
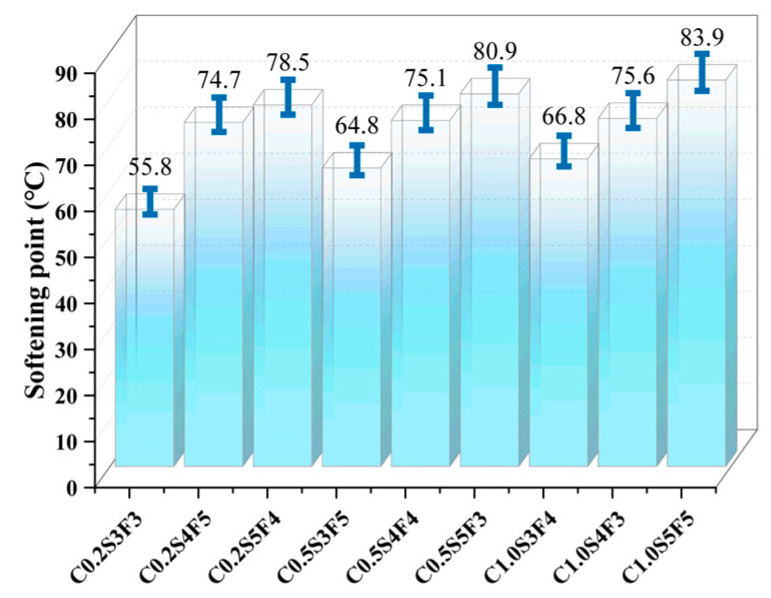
The softening point of modified sealants under the control of different dosage factor levels.

**Figure 7:**
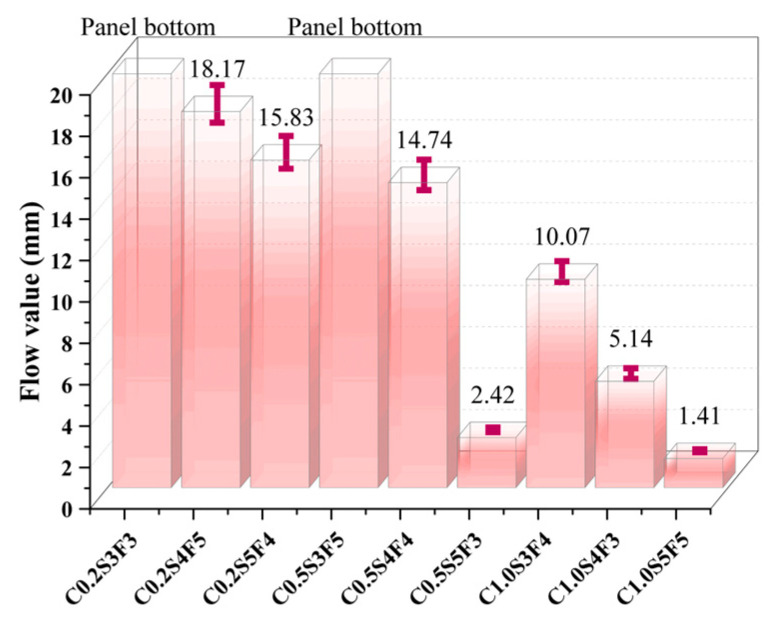
The flow value of modified sealants under the control of different dosage factor levels.

The softening point and flow value are both indicators in the evaluation of the high-temperature performance of sealants. The results of the softening point and flow value tests in this study are shown in Figure 6 and Figure 7. The high softening point and low flow value indicate that the modified sealant had excellent high-temperature stability, thus ensuring its performance in high-temperature environments and avoiding problems, such as run-off and sticky wheels. This leads to the conclusion that C1.0S5F5 and C0.5S5F3 had the best high-temperature performance in the orthogonal experimental group.

It can be seen in the range analysis in Table 6 that SBS had the greatest effect on the softening point of the sealant as well as on the flow value, far exceeding the effect of furfural oil extraction. This means that SBS occupied a dominant role in the sealants’ high-temperature performance improvement. This is the reason the softening point and flow values of the two groups of sealants, C1.0S5F5 and C0.5S5F3, were similar. Furfural-extracted oil is a lightweight component, and as an additive, it does not directly affect the performance of the potting sealant. Its main role is to improve the solubility of the SBS modifier in asphalt, thus improving the performance of the potting sealant. Therefore, the subsequent analysis of the performance of the sealant did not consider the influence of the furfural-extracted oil.

The asphalt modified by SBS did not change itself or the chemical structure of the asphalt molecule unit, and the modification process was mainly physical modification. The sufficient solubilization of asphalt components into polymer particles and good adsorption of polymer particles to asphalt components are the basis for the polymer modification of asphalt and the improvement of asphalt properties. This dynamic adsorption process induces a spatial three-dimensional mesh structure of SBS-modified asphalt, providing elasticity and improving the high-temperature performance of sealants. The increase in CNTs hardens the asphalt and improves the high-temperature performance of sealants. According to the softening point and flow value orthogonal experiments and the proposed SBS/CNT-modified asphalt sealant modifier addition program, 1 wt% CNT doping, 5 wt% SBS doping, 5 wt% furfural-extracted oil were used. Considering the economic efficiency of the material, the focus was on comparing the effect of CNTs on the performance of the sealants, so another sealant with a smaller amount of CNTs was chosen as the test group. Therefore, the C1.0S5F5 and C0.5S5F3 sealants were selected for subsequent testing in parallel with commercially available sealants MS1 and MS2 to compare their high-temperature rheological properties and aging resistance.

**Figure 8:**
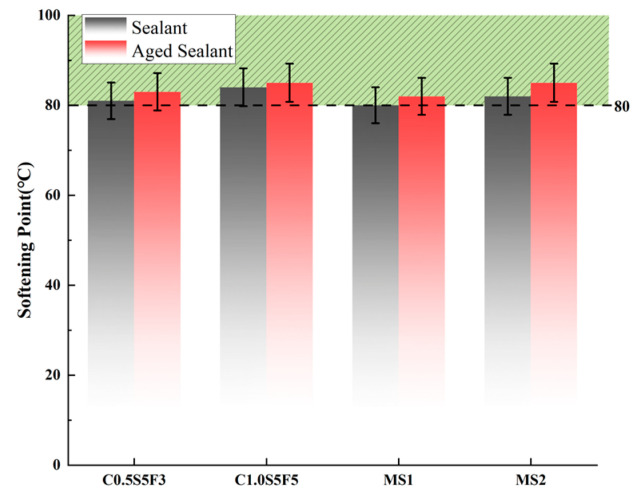
Comparison of softening point before and after aging.

**Figure 9:**
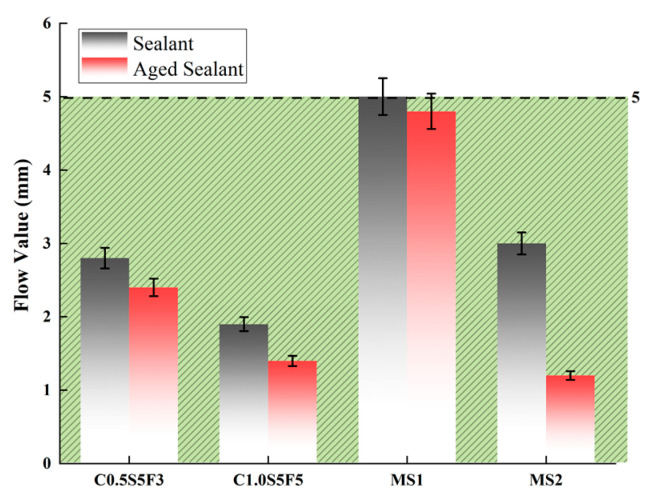
Comparison of flow value before and after aging.

The results of the C1.0S5F5, C0.5S5F3, MS1, and MS2 softening point tests and flow value tests are shown in Figure 8 and Figure 9, where the green area surrounded by the dashed line indicates the range of conformity indicators for the standard sealants. It can be seen that the softening points and flow values of the four types of sealants before and after aging meet the specification standards. The softening point and flow value of the sealants changed in opposite trends after aging; the softening point increased and the flow value decreased.

As can be seen in Figure 8, C1.0S5F5 had the largest softening point value, as it had higher CNT content, which means that the CNTs improved the high-temperature stability of the sealant [26]. Observing the increase in the softening point of the four types of sealants after aging, we can see that MS2 had the largest increase in the softening point after aging and was less resistant to thermal aging. Figure 9 shows that C1.0S5F5 had the smallest flow value, indicating that flow was least likely to occur under high-temperature conditions. MS1 had a larger flow value and was not sufficiently stable at high temperatures. After aging, MS2 had the largest drop in its flow value, a result that fits with the softening point results.

### 3.2. Rheological Property Analysis Based on Frequency Sweep Test

#### 3.2.1. Rutting Factor and Fatigue Factor

Dynamic mechanical testing of the sealants should be carried out within its linear viscoelastic range (LVE), and therefore, stress–strain scanning tests were carried out on the sealant specimens prior to the tests to ensure that the experimentally obtained rheological parameters were in accordance with linear viscoelastic theory [27]. The test parameters are shown in Table 7.

**Figure 10:**
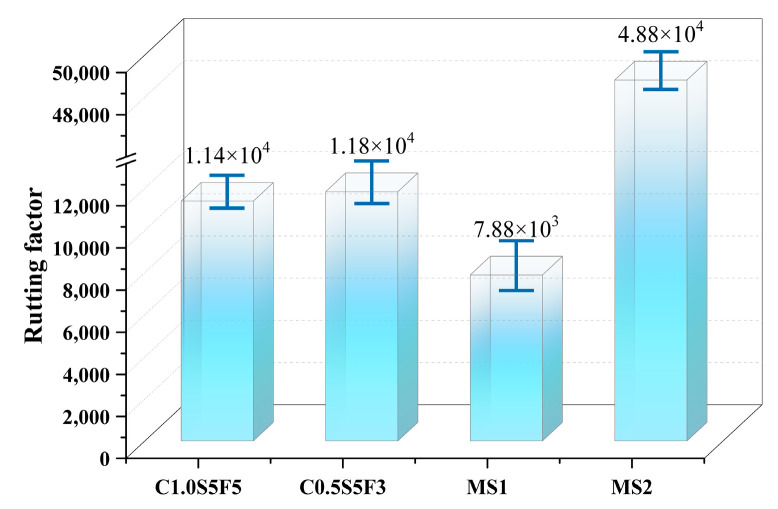
Comparison of rutting factors of four modified sealants.

The technical specifications for Superpave construction propose a rutting factor to assess the rutting resistance of asphalt materials at high temperatures. The rutting factor (G/Sinδ) is calculated from the complex shear modulus G and the phase angle *δ*, and a higher rutting factor means that the asphalt material is more resistant to rutting [28,29,30]. The calculated rutting factors of the four sealants in this study at 60 °C are shown in Figure 10.

Figure 10 shows that MS2 had the largest rutting factor, MS1 had the smallest rutting factor, and C1.0S5F5 and C0.5S5F3 had rutting factors between the first two. It can be concluded that the rutting resistance of SBS/CNT-modified asphalt sealants can exceed that of commercially available sealants, but there is still room for improvement. The higher the amount of CNTs added, the better the rutting resistance of the sealants. The rutting factor of C1.0S5F5 was smaller than that of C0.5S5F3. This indicates that there is an optimum amount of CNTs to be added to improve the high-temperature rutting resistance of the sealant.

**Figure 11:**
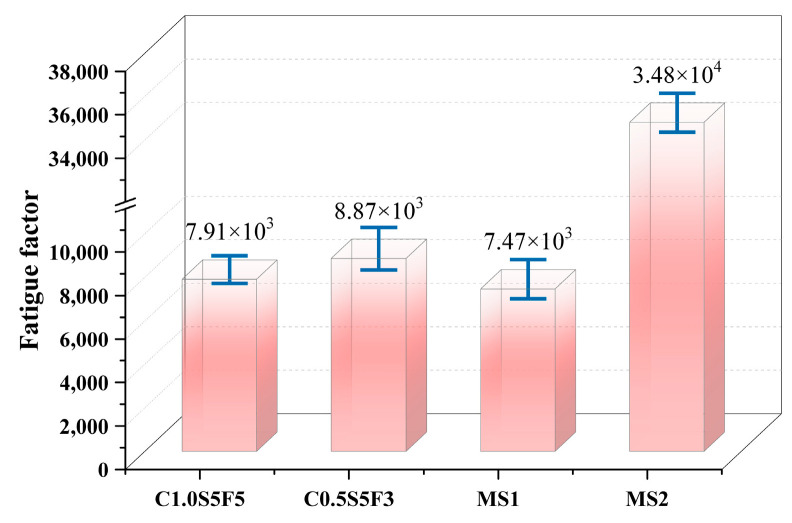
Comparison of fatigue factors of four modified sealants.

The fatigue factor is an indicator evaluating the fatigue cracking resistance of asphalt materials. The fatigue factor (G·Sin) is calculated from the complex shear modulus *G* and the phase angle *δ*. The smaller the fatigue factor, the greater the elasticity and resistance to fatigue cracking of bituminous material [31,32,33]. The elasticity and fatigue resistance of sealants, which are exposed directly to the natural environment after construction and are subject to weather and vehicle loads, are of paramount importance, so the fatigue factors of the four sealants were calculated, and the results are shown in Figure 11.

As can be seen in Figure 11, MS2 had the largest fatigue factor, MS1 had the smallest fatigue factor, C1.0S5F5 was about the same as MS1, and C0.5S5F3 had a slightly higher fatigue factor than C1.0S5F5. This indicates that MS2 had the weakest fatigue cracking resistance, while C1.0S5F5 and MS1 had relatively higher fatigue cracking resistance. It can be seen that the addition of CNTs had an obvious effect on the fatigue resistance of the sealants, which can play a toughening role in sealants and a positive role in improving the fatigue life of sealants. Despite its high-temperature performance, MS2 is less resistant to fatigue and more susceptible to fatigue cracking with increasing road use.

#### 3.2.2. Energy Storage Modulus and Dissipation Modulus

The energy storage modulus $G^{\prime }$ and the dissipation modulus $G^{\prime \prime }$ are the real and imaginary parts of the complex shear modulus *G*, respectively, as in Equation (1). $G^{\prime }$ is the energy stored and released by the asphalt material under stress and reflects the elastic component of the asphalt material. $G^{\prime \prime }$ is the energy lost in the form of heat by friction within the sealant during the test and reflects the viscous component of the material [34]. Further analysis of the elastic and viscous behavior of the sealants under different temperature and frequency conditions by $G^{\prime }$ and $G^{\prime \prime }$.

(1) $$G=\frac{\tau }{\gamma }=G^{\prime }+iG^{\prime \prime }=\left|G\right|\cdot cos\delta +i\left|G\right|\cdot sin\delta$$

**Figure 12:**
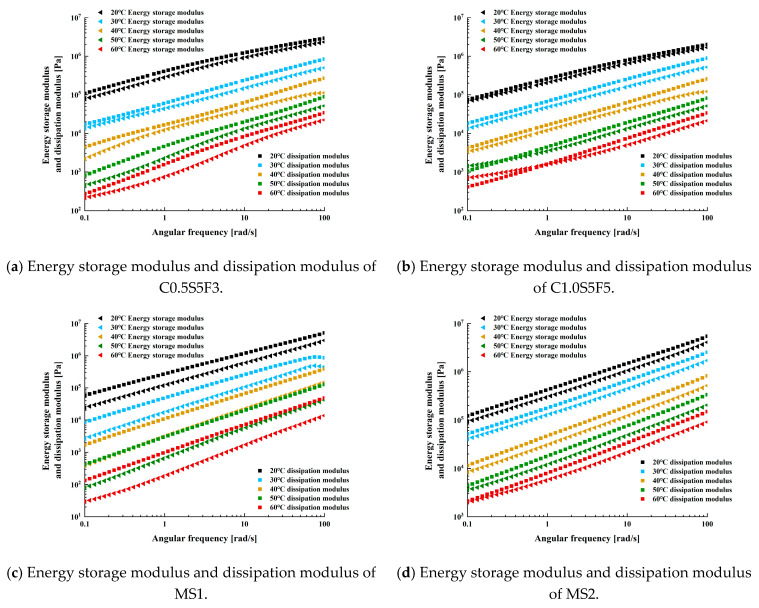
Variation in energy storage modulus and dissipation modulus with frequency for different temperatures of sealants.

Figure 12 shows the variation in the energy storage modulus and dissipation modulus with frequency for the four types of sealants in different temperature conditions. It can be seen that both the $G^{\prime }$ and $G^{\prime \prime }$ of each sealant had a significant increase with increasing frequency, with an increase of up to two orders of magnitude in the frequency range of 0.1 to 100 rad/s. In the range of the frequencies tested, $G^{\prime \prime }$ was higher than $G^{\prime }$ overall, indicating that the rheology of several sealants was influenced by the viscous behavior. In addition to frequency, $G^{\prime }$ and $G^{\prime \prime }$ were also influenced by the composition of the sealants. The difference between $G^{\prime }$ and $G^{\prime \prime }$ in the variation pattern of C1.0S5F5 was significantly smaller than that of the other three sealants. When the temperature was 20 °C and 30 °C, $G^{\prime }$ and $G^{\prime \prime }$ appear to intersect, gradually increasing. This shows that the elastic behavior of the C1.0S5F5 sealant increased gradually with increasing frequency at room temperature. At the same time, the CNT content in C1.0S5F5 was higher compared to that in C0.5S5F3, which had a higher $G^{\prime }$ and $G^{\prime \prime }$ than C0.5S5F3, which was also higher than in the MS1 sealant. This is related to the SBS/CNT-modified asphalt sealant system, in which CNTs enhance the diffusion of the light components of the asphalt phase and promote the swelling of the SBS phase, resulting in the formation of a denser polymer network [35].

#### 3.2.3. Rheological Master Curve Fitting Based on CAM Model

The time–temperature equivalence principle assumes that the temperature scale and the frequency scale can be converted equivalently. The rheological master curve established by the time–temperature equivalence principle enables the viscoelastic characteristics of the material to be obtained over a wide frequency range [36]. It can be expressed as the WLF formula, from which the horizontal shift factor $\alpha_{T}$ can be calculated, as shown in Equation (2):

(2) $$lg\alpha_{T}=\frac{-C_{1}\left(T-T_{0}\right)}{C_{2}+\left(T-T_{0}\right)}$$

where $lg\alpha_{T}$—shift factor; $C_{1}$, $C_{2}$—constant; T—test temperature, °C; $T_{0}$—reference temperature, °C.

**Figure 13:**
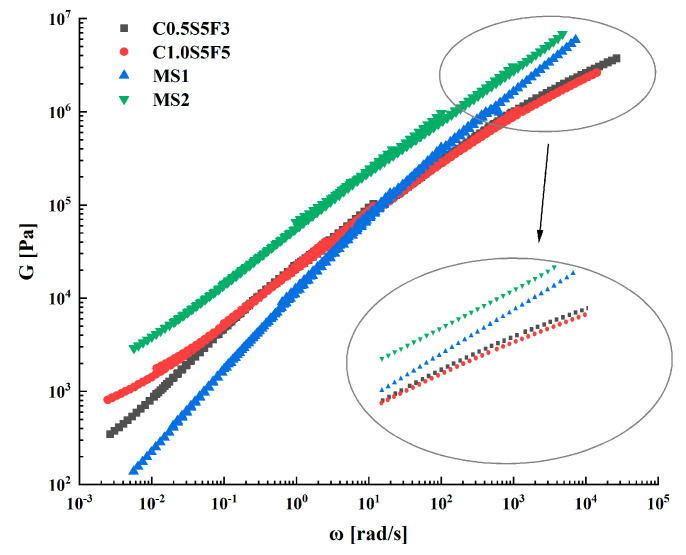
The master curve of the complex modulus of the four sealants.

Figure 13 shows the master curve of the complex modulus of the four sealants in the double logarithmic axis at 40 °C. The main curves of the sealants had a similar trend, with the highest modulus values at high frequencies (low temperatures), and the same response characteristics as the actual pavement. In the low-frequency (high-temperature) region, the complex modulus master curve of C1.0S5F5 is higher than those of C0.5S5F3 and MS1. However, there is a crossing point in the frequency domain of 10~100 rad/s for the three kinds of sealants, i.e., when the frequency exceeds the crossing frequency, the moduli of MS1 and C0.5S5F3 are larger than that of C1.0S5F5, and the difference became larger and larger with the increase in frequency. This phenomenon is attributed to the fact that the two sealants, MS1 and C0.5S5F3, gradually approached the glassy state as the temperature decreased, while the C1.0S5F5 sealant remained flexible. The C1.0S5F5 and C0.5S5F3 prepared in this study showed similar rheological behavior. The modulus difference between the two gradually decreased in the high-frequency (low-temperature) region, and the frequency dependence gradually decreased. The change in the dosage of CNTs had a greater influence on the viscoelastic behavior under low-frequency (high-temperature) conditions.

Overall, the slopes of the curves for the C1.0S5F5, C0.5S5F3, and MS2 sealants are close to each other and lower than the slope of MS1. This suggests that the frequency dependence of the three sealants is much lower. The SBS molecule and the long-chain alkane branched chains in the asphaltene are entangled with the CNTs’ tubular diameter, and the CNTs’ interfacial pulling action connects the asphaltene phase to the SBS phase, which results in a more stabilized internal structure of the material [13].

**Figure 14:**
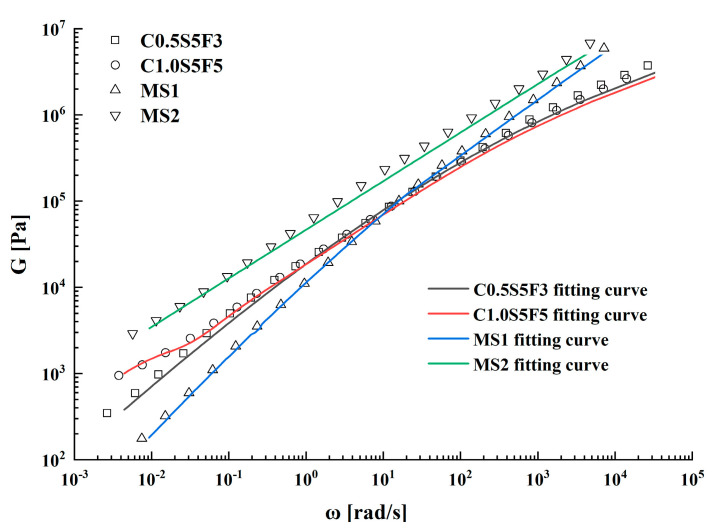
Master curve and fitted curve for complex shear modulus of sealants.

The main complex shear modulus curves for the four types of sealants reflect the differences in performance between the different types of sealants over a wide frequency range. However, due to the range of the equipment, these complex shear modulus master curves do not yet clearly reflect the differences in the moduli among the materials at extremely high and low frequencies, so a rheological model was fitted for analysis. The CAM model is commonly used for master curve fitting and follows Equation (3). The fitted curves for the complex modulus master curve based on the CAM model are shown in Figure 14:

(3) $$\left|G\right|=\frac{\left|G_{g}^{*}\right|}{{\left[{1+\left(\omega_{c}+\omega_{r}\right)}^{K}\right]}^{M/K}}$$

where $\omega_{r}$ is the frequency of the main curve, $\left|G_{g}^{*}\right|$ represents $\left|G\right|$ when converging to ∞, $\omega_{c}$ is the crossover frequency, and *M* and *K* are the shape parameters.

The fitting results for all sealants are shown in Table 8. The CAM model has a high correlation for fitting the complex modulus master curves of the four sealants, with an *R*^2^ of 0.999. A larger $\omega_{c}$ indicates more viscous components in the sealant. From the point of view of the road performance of the sealant, dynamic shear under the action of the rheology of the material by the viscous dominant means that it has a better relaxation property and can better avoid secondary cracking after the construction of the sealant.

### 3.3. Mechanical Properties of Sealants under Dynamic Load–Temperature Coupling Actions Based on MSCR Tests

#### 3.3.1. Non-Recoverable Compliance and Recoverable Creep

Creep will be formed in viscoelastic materials under stress. After the external force is removed, the elastic deformation recovers immediately, the delayed deformation recovers gradually, and the viscous deformation that cannot be recovered is called permanent deformation. This is where viscoelastic materials differ from viscous and elastic materials. After several loading cycles, the non-recoverable part of the material gradually accumulates to a cumulative strain, the smaller the cumulative strain, the better the material’s ability to recover from deformation. The deformation generated by the vehicle load is similar to this strain accumulation process, and the MSCR test simulates the repeated action pattern of loading–unloading of the vehicle load.

The creep recovery rate *R* and non-recoverable compliance $J_{nr}$ are the evaluation indicators for the MSCR test. *R* reflects the elastic deformation capacity of the sealants; the larger the *R*, the stronger the elastic deformation capacity of the material. $J_{nr}$ reflects the high-temperature deformation resistance of the sealants; the larger the value, the greater the irrecoverable deformation of the sealants. The formulas are shown in Equations (4) and (5):

(4) $$R=\frac{\gamma_{p}-\gamma_{nr}}{\gamma_{p}-\gamma_{0}}\times 100\%$$

(5) $$J_{nr}=\frac{\gamma_{nr}-\gamma_{0}}{\tau }$$

where $\gamma_{p}$ is the peak strain; $\gamma_{nr}$ is the residual strain; $\gamma_{0}$ is the initial strain; and τ is the creep shear stress, kPa. The final index is the average of 10 cycles at each stress level as the performance evaluation index of the sealants, as shown in Equations (6) and (7):

(6) $$\bar{R}=\sum_{n=1}^{10}R\left(\sigma ,n\right)/10$$

(7) $$\bar{J_{nr}}=\sum_{n=1}^{10}J_{nr}\left(\sigma ,n\right)/10$$

**Figure 15:**
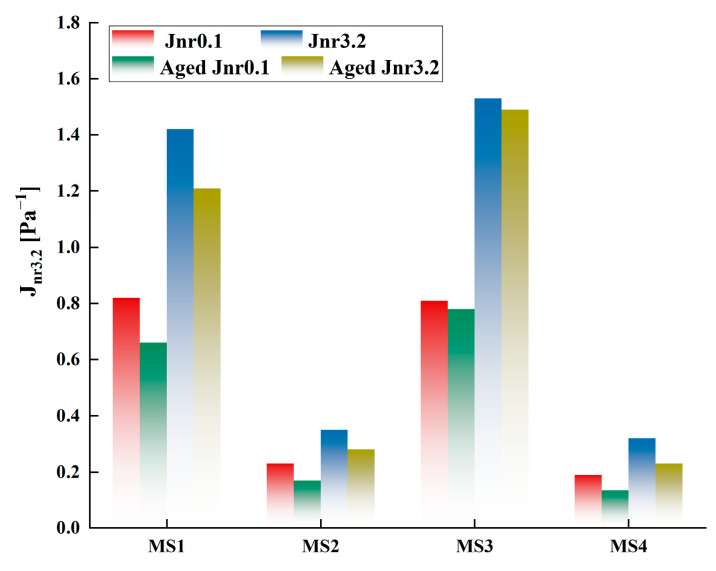
$J_{nr}$ of four kinds of modified sealants before and after aging under different stress conditions.

**Figure 16:**
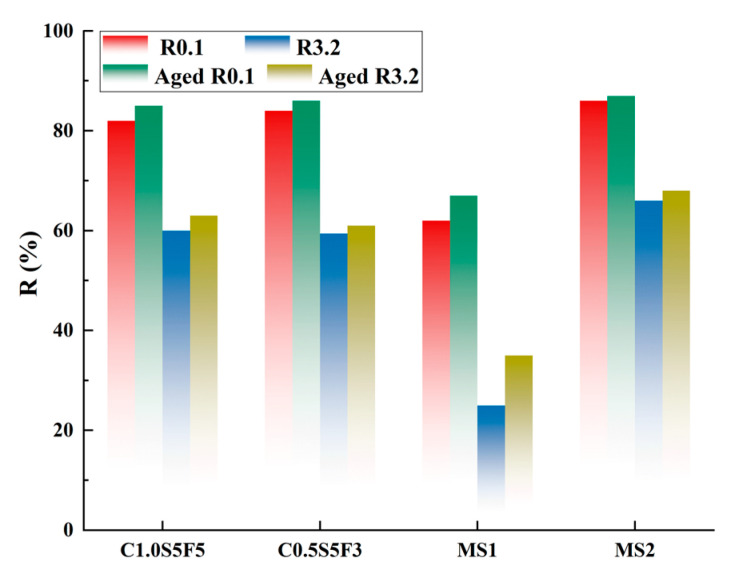
*R* of four kinds of modified sealants before and after aging under different stress conditions.

The results of calculating the $J_{nr,0.1}$, $J_{nr,3.2}$, $R_{0.1}$, and $R_{3.2}$ indicators of the four types of sealants before and after aging are shown in Figure 15 and Figure 16. The $J_{nr}$ of all four sealants under different stress conditions decreased after heating and aging, which means that the irrecoverable deformation of the sealants under load decreased. This also indicates that the hardening effect of aging on the sealants made them exhibit better resistance to deformation at high temperatures [37]. The C1.0S5F5 sealant had the most significant decrease in the $J_{nr}$ indicator after aging, while the C0.5S5F3 and MS2 sealants had smaller $J_{nr}$. The high-temperature deformation resistance of C1.0S5F5 was superior because CNTs act as short-fiber reinforcement between asphalt and SBS. In addition, the *R*-values of the sealants became larger after aging, which, according to their physical significance, indicates that the elastic recovery properties of the aged sealants were enhanced. This is attributed to the fact that the sealants hardened under the influence of thermal aging and became less adhesive. The MS1 sealant had the smallest *R*-value, with the remaining three having close *R*-values.

Comparing the results at different stress, agitation, heating, and aging levels reduced the stress sensitivity of the sealants $J_{nr}$ and *R*. This also indicates an increase in the high-temperature deformation resistance of the sealants due to aging and the presence of a hardening effect of the sealants [38]. As a crack-filling and repair material, the bonding and elasticity of sealants are crucial. A combination of parameters is needed to examine the changes in the performance of sealants after aging. Even if the non-recoverable compliance of the sealant decreases after aging, it cannot simply be assumed that aging has improved its high-temperature performance. It should also be noted that aging leads to a decrease in the elastic recovery index of sealants. It is also necessary to consider its ability to resist the effects of thermal aging and to assess the stability of the performance of sealants during construction.

#### 3.3.2. Burgers Model Fits Creep Curves of Sealants

**Figure 17:**
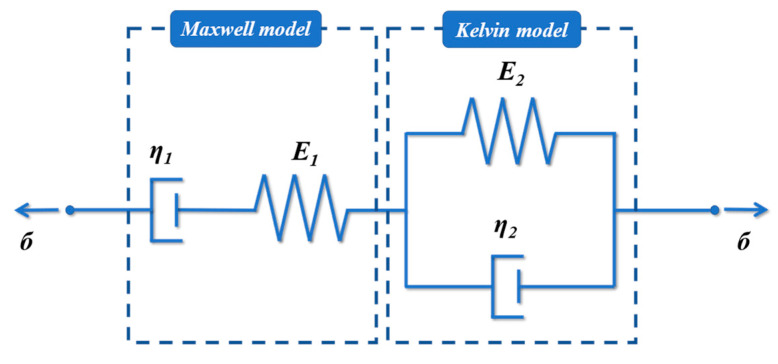
Burgers model.

**Figure 18:**
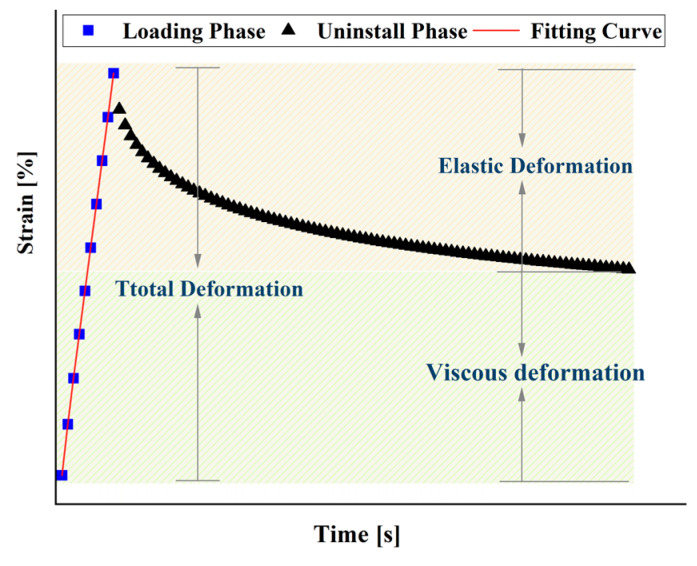
Burgers model—creep test fitting diagram.

The Burgers model is a widely used model for viscoelastic mechanics. It is a four-element model consisting of a Kelvin model and a Maxwell model in a series, considering the advantages of both the Kelvin and Maxwell models, as shown in Figure 17. Figure 18 shows the creep curve for a single cyclic cycle, which was 1 s for the loading phase and 9 s for the unloading phase. The Burgers model was fitted to the loading phase.

The Burgers fitting equation used in this paper is shown in Equation (8):

(8) $$\varepsilon =\tau_{0}\left[\frac{1}{E_{1}}+\frac{1}{\eta_{1}}+\frac{1}{E_{2}}\left(1-e^{-E_{2}t/\eta_{2}}\right)\right]$$

where $\tau_{0}$ is the constant shear stress. $E_{1}$ and $\eta_{1}$ are the elasticity modulus and viscosity coefficient in the Maxwell model, which characterize the elastic and viscous deformation of the material respectively. $E_{2}$ and $\eta_{2}$ are the elasticity modulus and viscosity coefficient in the Maxwell model, respectively, which reflect the creep properties of the material.

The classical Burgers model was fitted for the C1.0S5F5, C0.5S5F3, MS1 and MS2 sealant creep comparisons, and the fitting results are shown in Table 9.

As can be seen in Table 9, the Burgers model fits well with the creep loading stage, and the *R*^2^ reaches more than 0.998. $E_{1}$ represents the instantaneous modulus of elasticity. The value of $E_{1}$ of the C1.0S5F5, C0.5S5F3, and MS1 sealants increased after aging, reflecting that their resistance to elastic deformation was enhanced after aging.

$\eta_{1}\;$ reflects the irrecoverable deformation viscosity coefficient. The larger $\eta_{1}\;$ is, the smaller the permanent deformation produced by the sealant in the same amount of time. Under 0.1 kPa stress, the $\eta_{1}\;$ of the sealants increased after aging, that is, the four kinds of sealants are more likely to produce permanent deformation under this stress. Under 0.1 kPa stress, C1.0S5F5 had the largest $\eta_{1}.$ This indicates that under heavy traffic conditions, C1.0S5F5 will produce minimal permanent deformation and has excellent resistance to deformation. This is because CNTs can deepen the degree of swelling of SBS, making the SBS and asphalt components fully crosslinked. The SBS network becomes denser, increasing the elasticity of the asphalt and improving the road performance of the asphalt [39].

## 4. Conclusions

In this study, a modified asphalt sealant was prepared by adding SBS and CNTs to asphalt. Firstly, a preliminary evaluation of the high-temperature performance of sealants was carried out. Secondly, the high-temperature rheology and aging resistance of the sealants were further investigated by dynamic shear rheology tests, and the material ratios for the preparation of the sealants were determined by comprehensive evaluation, using 1 wt% CNTs, 5 wt% SBS, and 5 wt% furfural-extracted oil. The creep process of the sealants was then fitted by the CAM model to the main curve and the Burgers model to better understand the dynamic viscoelasticity of the modified sealants. The main conclusions reached are as follows:

(1) By comparing the softening point and flow value, C1.0S5F5 and C0.5S5F3 showed excellent high-temperature performance before and after aging compared to the ordinary commercially available sealants. Based on the range analysis, SBS and CNTs play a staple role in strengthening the heat resistance of sealants.
(2) The SBS/CNT-modified asphalt sealants were more resistant to rutting than the commercially available sealants, and the addition of CNTs had a more obvious effect on the fatigue resistance of the sealants and can play a toughening role in sealants.
(3) The CAM model fitting results show that the model correlates well with the modulus curve and can be used as an effective method for characterizing and predicting the viscoelastic behavior of sealants. The addition of CNTs can increase the glassy composite shear modulus and rheological index of asphalt, thereby increasing the shear strength and reducing the temperature sensitivity of asphalt. C1.0S5F5 had a better relaxation property, which can better avoid secondary cracking after the construction of the sealant.
(4) According to the MSCR test, the high-temperature deformation resistance of the C1.0S5F5 sealant before aging was better, and the elastic recovery ability of the C1.0S5F5 and C0.5S5F3 sealants was significantly stronger than that of the MS1 sealant. CNTs act as short-fiber reinforcement between asphalt and SBS.
(5) The correlation coefficients fitted using the Burgers model were all greater than 0.99, which is a good predictor of the viscoelastic intrinsic characterization of the sealants. With the Burgers model, C1.0S5F5 showed excellent deformation resistance under heavy traffic conditions.

Based on the above conclusions, SBS/CNTs-modified asphalt sealant can not only meet the high-temperature performance required in the standard, but also has excellent thermal stability and fatigue properties due to the modification of CNTs and SBS. Based on these conclusions, the findings of the study can also provide experimental and theoretical support for the popularization of SBS/CNT-modified asphalt sealant applications.

## Figures and Tables

**Table 1:** The fundamental properties of 90# matrix asphalt.

Index	Unit	Standard Value	Measured Results
Penetration (25 °C, 100 g, s)	0.1 mm	80~100 (0.01 mm)	84 (0.01 mm)
Softening Point (Global Method)	°C	≥45 (°C)	46.0 (°C)
Ductility (15 °C)	cm	≥20 (cm)	>100 (cm)

**Table 2:** The fundamental properties of CNTs.

Inner Diameter (nm)	Outer Diameter (nm)	Length (µm)	Specific Surface Area (m^2^/g)	Purity (%)	Grain Size D50 (µm)
3–5	8–15	8–14	≥250	>99	≤25

**Table 3:** The fundamental properties of SBS.

Shape	Styrene/Butadiene	Tensile Strength (MPa)	Molecular Weight (×10,000)
Star	40/60	26	16 ± 2

**Table 4:** Three factors and three levels.

Factors	Levels		
	CNT Content (wt%)	SBS Content (wt%)	Furfural-Extracted Oil Content (wt%)
1	0.2	3	3
2	0.5	4	4
3	1.0	5	5

**Table 5:** Orthogonal scheme design.

Test Number	CNT Content (wt%)	SBS Content (wt%)	Furfural-Extracted Oil Content (wt%)	Group No.
1	0.2	3	3	C0.2S3F3
2	0.2	4	5	C0.2S4F5
3	0.2	5	4	C0.2S5F4
4	0.5	3	5	C0.5S3F5
5	0.5	4	4	C0.5S4F4
6	0.5	5	3	C0.5S5F3
7	1.0	3	4	C1.0S3F4
8	1.0	4	3	C1.0S4F3
9	1.0	5	5	C1.0S5F5

**Table 6:** Extreme variance analysis table for each factor for each indicator.

Evaluation Indicators	Level of Factors	CNT Content	SBS Content	Furfural-Extracted Oil Content
Softening point (°C)	k_1_	69.7	62.5	70.8
	k_2_	73.6	75.1	73.4
	k_3_	75.4	81.1	74.4
	Extreme variance	5.7	18.6	3.6
Flow value (mm)	k_1_	24.7	30.0	15.9
	k_2_	19.0	12.7	13.5
	k_3_	5.5	6.6	19.9
	Extreme variance	19.2	23.4	6.0

**Table 7:** Frequency sweep test parameters.

Specimen Number	Test Temperature	Parallel Plate Diameter	Thickness	Frequency Range	Loading Strain
C0.5S5F3, C1.0S5F5, MS1, MS2	20 °C, 30 °C	8 mm	2 mm	0.1~100 rad/s	Within the linear viscoelastic range

**Table 8:** Fitting viscoelastic parameters results of CAM model.

Sample	$\omega_{c}$/Hz	*M*	*K*	*R* ^2^
C1.0S5F5	1837.3	0.8749	0.1468	0.999
C0.5S5F3	1338.8	0.9205	0.1795	0.999
MS1	1303.6	1.3042	0.0696	0.999
MS2	2942.1	1.0936	0.0488	0.999

**Table 9:** Fitting viscoelastic parameters results of Burgers model.

	Stress								*R* ^2^
	0.1 kPa				3.2 kPa				
Sample	$E_{1}$/kPa	$\eta_{1}$/kPa·s	$E_{2}$/kPa	$\eta_{2}$/kPa·s	$E_{1}$/kPa	$\eta_{1}$/kPa·s	$E_{2}$/kPa	$\eta_{2}$/kPa·s	
C1.0S5F5	0.0145	0.00663	0.0378	0.0227	1.33	243.18	7.87	30.67	0.99876
C0.5S5F3	0.0028	0.0176	0.0791	0.0272	0.0017	0.0113	0.6761	0.0616	0.99812
MS1	1.02	19.28	81.35	20.76	7.57 × 10^−2^	11.60	7.51 × 10^−2^	3.08	0.99942
MS2	24.31	140.75	135.68	34.28	20.06	114.98	497.78	36.62	0.99911
After aged									
C1.0S5F5	0.91	4.97	66.16	11.81	1.33	243.18	7.87	30.67	0.99945
C0.5S5F3	0.0081	0.0230	0.00627	0.0218	0.0024	0.2270	0.0125	0.0474	0.99873
MS1	1.32	21.59	100.41	27.59	0.096	10.34	0.94	2.81	0.99905
MS2	15.12	756.87	159.09	26.62	31.49	107.26	381.59	32.81	0.99892

## Data Availability

The data presented in this study are available upon request from the corresponding author.
